# Qualification of the PAVIN Fog and Rain Platform and Its Digital Twin for the Evaluation of a Pedestrian Detector in Fog

**DOI:** 10.3390/jimaging9100211

**Published:** 2023-10-03

**Authors:** Charlotte Segonne, Pierre Duthon

**Affiliations:** Intelligent Transportation System Research Team, Cerema, 63100 Clermont-Ferrand, France

**Keywords:** pedestrian detection, digital twin, fog, simulation

## Abstract

Vehicles featuring partially automated driving can now be certified within a guaranteed operational design domain. The verification in all kinds of scenarios, including fog, cannot be carried out in real conditions (risks or low occurrence). Simulation tools for adverse weather conditions (e.g., physical, numerical) must be implemented and validated. The aim of this study is, therefore, to verify what criteria need to be met to obtain sufficient data to test AI-based pedestrian detection algorithms. It presents both analyses on real and numerically simulated data. A novel method for the test environment evaluation, based on a reference detection algorithm, was set up. The following parameters are taken into account in this study: weather conditions, pedestrian variety, the distance of pedestrians to the camera, fog uncertainty, the number of frames, and artificial fog vs. numerically simulated fog. Across all examined elements, the disparity between results derived from real and simulated data is less than 10%. The results obtained provide a basis for validating and improving standards dedicated to the testing and approval of autonomous vehicles.

## 1. Introduction

Certification up to SAE level 3 is now possible for vehicles featuring partially automated driving [1]. The manufacturer must demonstrate that its vehicles ensure adequate safety conditions within their operational design domain (ODD), having conducted tests in diverse scenarios [2]. Artificial intelligence (AI) is mostly used for automated tasks (e.g., visual or mixed navigation, sign recognition, road tracking, obstacle detection). The qualification of these systems requires verification in all kinds of scenarios, taking into account adverse weather conditions. For cost and safety reasons, all of these qualification tests cannot be carried out in real conditions, as some tests may present risks or have frequencies of occurrence that are too low to allow for the collection of large series of data. For this reason, sensors and simulation tools for adverse weather conditions (physical, numerical, or hybrid) must be implemented.

Pedestrian detection is a fundamental task in the deployment of autonomous vehicles (AVs) within cities, as pedestrians are vulnerable road users (VRUs). In particular, this task is the first braking-related advanced driver assistance system (ADAS) that has been implemented as an “Automatic Emergency Braking” (AEB) [3].

Regarding the validation of a pedestrian detector using AI, many research questions arise:

- First, how can we guarantee that we have tested a wide enough range of conditions? AI-based algorithms are black boxes and it is, therefore, very difficult to find their boundary conditions. Indeed, the typology, position, and orientation of the pedestrian can influence the results of the algorithm. Similarly, the environment, disturbing objects, and occlusions can influence the detection. Beyond these geometric issues, weather conditions also have strong impacts, e.g., illumination, camera glare [4,5], fog [6,7,8,9,10], rain [7,10,11], and snow [8]. Interest in this issue is recent in the field of autonomous vehicles and is the subject of numerous studies [12,13,14], but at present, the works listed in the literature only present particular cases and not a global solution.
  Even if all the conditions required for successful validation have been identified, it is impossible to reproduce them all in real-world conditions. For this, one solution is to use numerical simulation. Many numerical simulators dedicated to autonomous vehicles exist [15,16,17,18]. Most offer variants regarding pedestrians, environments, or weather, but only a few are calibrated [15] against real-world conditions, to our knowledge.
- The second question is: How can we validate the realism and representativeness of a digital simulator? Will the behavior of artificial intelligence be the same in front of the simulator and in reality? To make things more complex, the data can be partially or totally simulated, so X-in-the-loop simulators appear with augmented reality mechanisms. It is a model of this type that we propose to test in this article.
  Beyond numerical simulation, physical simulation methods are used to simulate adverse weather conditions. This is the case with the PAVIN fog and rain platform, which can reproduce adverse weather conditions on demand [19]. This platform is calibrated from a meteorological point of view (calibration of intensities, drop size, and velocity). However, unlike a purely simulated test, a physical test must be qualified from a repeatability point of view. This is essential in the context of certification tests, where test laboratories are often qualified and audited, making repeatability tests and uncertainty measurements mandatory. Can this type of platform guarantee the repeatability of tests, as well as a standard deviation on the results obtained with AI?

In an attempt to answer these questions, this paper introduces a new pedestrian database, focusing on weather and lighting variations (day, night, clear weather, and fog) and an associated evaluation method of detection tools. The Cerema Foggy-Twin Pedestrian (CFTP) database comprises real data, gathered in clear weather and artificial fog conditions within the PAVIN fog and rain platform, and also numerically simulated data (digital twin), executed in the hardware-in-the-loop mode, from a simplistic model prevalently used in most numerical simulators outlined in existing literature. Both real and simulated data are annotated with 2D pedestrian detection bounding boxes.

This work presents different original contributions. First of all, in the literature, databases for autonomous vehicles are either focused on adverse weather conditions but without pedestrians in the scenes, or on the presence of pedestrians but without—or not enough—variation in the weather conditions [8,20,21,22,23,24,25]. Moreover, databases contain either real data or simulated data, but rarely both. On the contrary, our CFTP database combines all these aspects, which includes both real data and data from numerical simulations, with various pedestrians in different weather and lighting environments. In [26], a table lists the different elements of diversity in the main databases for autonomous driving (Waymo [21], BDD100k [27], nuScenes [22], ONCE [26]), in terms of weather (sunny, rainy, cloudy, foggy, ...), area of interest (city, tunnel, bridge, etc.), or time of day (day, night, dawn). The only database with foggy images is BDD100k, but they make up only 0.18% of the database. All these databases contain rainy weather images, but again, the proportion of such data is low (from 0.6% for Waymo and up to 19.43% for nuScenes) compared to clear weather conditions. Even though those databases contain a wide variety of real-life scenes, ours focuses on a single urban scene recreated in the PAVIN fog and rain platform and provides diversity in the number of parameters, such as weather, lighting, and pedestrian variability (clothing, position, height, etc.) for both clear and foggy conditions, with equal data numbers for each weather condition.

Secondly, the quality and representativeness analysis of the standard of pedestrian detection tools (e.g., AEB) for autonomous vehicle systems is not well-documented in the literature. Indeed, the standard ISO/DIS 22733–2 [3] suggests to test those conditions using a unique certified dummy, with no variation in size, clothing, or color. In addition, the test conditions are also fixed (identical route, clear weather conditions only). The method proposed in this paper, associated with the database produced, allows us to analyze the variations of results of an algorithm, according to various criteria, such as the typology of the pedestrian and their position. It also allows checking how many different pedestrians or images are necessary to produce a wide enough database to validate an AI-based algorithm, guaranteeing a minimal error in the results.

Finally, this work aims to verify the representativeness and realism of the output images of a widely used fog model. This model, called the Koschmieder model [28], consists of reproducing the visual effect of fog on fog-free images by applying a reduction of contrast. It is commonly used in today’s simulators [16,17,18], but some studies show that it has important limitations [15]. This type of validation remains rare in the literature [15]. The Koschmieder model is used here in the hardware-in-the-loop mode, using the images of the camera without fog, to add fog to it.

The remainder of this paper is organized as follows: Section 2 presents a method to check the quantitative quality of a database, whether it is derived from real or simulated data. Section 3 presents the process of database creation, from two methods of fog production: artificially reproduced and numerically simulated. Finally, Section 4 presents the results obtained and discussions held throughout this work, followed by several conclusions and prospects for future work.

## 2. Method

As previously explained, vehicle certification will bypass the use of physical test facilities and numerical simulations to address adverse weather conditions, such as fog. The objective of this paper is plural. The idea is to validate a physical test facility together with a hardware-in-the-loop (HiL) numerical simulator for the qualification of AI-based pedestrian detectors. The physical test facility is the PAVIN fog and rain platform and the numerical fog simulator is the Koschmieder law-based simulator developed by Cerema, called the K-HiL simulator, in the following. At the same time, it is necessary to validate the associated protocol to be implemented for this type of qualification and identify the volume of data required, e.g., the variety of pedestrians, the number of images, and the stability of the fog. To this end, rather than focusing on the raw sensor images, this work proposes a method based on the analysis of the final scores obtained by a classical artificial intelligence-based pedestrian detection algorithm [29].

**Figure 1:**
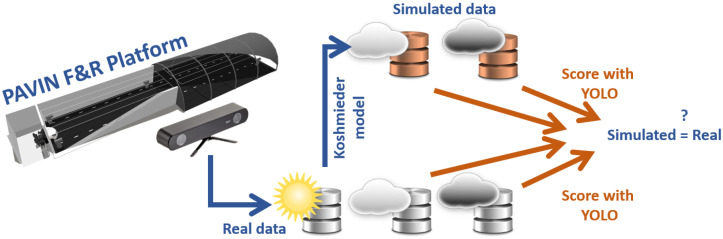
The principle of the method used to compare physical and numerical fog simulation.

Figure 1 presents the proposed method in a general way. This method is based on a database of labeled pedestrian images, a classical pedestrian detection algorithm [29], and a metric to evaluate this algorithm (area under the precision and recall curve, AUC). Finally, the YOLO algorithm is applied to the whole database. The scores are compared to check the similarities.

Regarding the database, it contains real images of pedestrians in daytime conditions under three weather conditions (clear weather, medium fog, and dense fog) acquired by a camera within the PAVIN fog and rain platform. Then, a numerically simulated fog (K-HiL) is added to the images acquired in clear weather, as shown in Figure 1. This allows us to verify if the results obtained by the detection algorithm on the two databases are similar. For this purpose, we propose checking two fog levels, medium and dense fogs (MF and DF) and clear weather conditions (CW).

**Figure 2:**
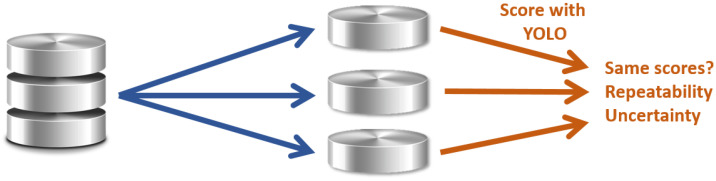
Method to define the minimum characteristics of a pedestrian detector validation database.

Similarly, our second objective in this study is to verify the repeatability of the scores obtained and the accuracy of an algorithm through a database, depending on the characteristics of the latter. This is essential for certification, which requires qualified testing means. We, therefore, propose analyses of different subsets from the database according to different criteria (type of pedestrian, number of pedestrians, number of images, variability of fog, accessory size, etc.) (Figure 2). Thus, the detection algorithm is applied to each subset. The scores are compared to check the similarities. It allows us to identify the minimum amount of data to be produced to guarantee results with an associated uncertainty.

The database, therefore, contains two types of data in foggy conditions: a fog artificially produced in an indoor facility and a numerically simulated fog. Before describing the method in more detail, these two ways of producing fog are described throughout the rest of this section.

### 2.1. Physically Simulated Fog: PAVIN Fog and Rain Platform

**Figure 3:**
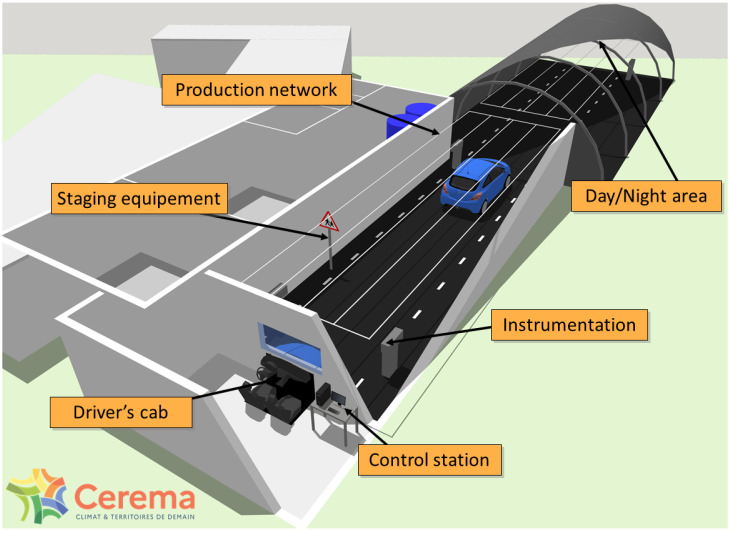
PAVIN fog and rain platform scheme.

The artificial fog is produced in the PAVIN fog and rain platform [19]. It allows the production of various and reproducible fog and rain conditions. The PAVIN fog and rain platform is a facility situated in Clermont-Ferrand (France). The platform dimensions are as follows: 30 m long, 5.5 m wide, and 2.20 m high. Its dimensions allow the reproduction of an urban scene and, thanks to a removable greenhouse, it is also possible to reproduce day or night conditions on this platform [19]. Figure 3 shows a scheme of the platform. Only the “Day and Night area” on the upper part of Figure 3 has been used to create the urban scene. This part of the platform is 18 m long and approximately 8 m wide.

Fog is characterized in meteorology by the Meteorological Optical Range (MOR), also called visibility, and noted as *V* [30]. MOR, expressed in meters, corresponds to the distance at which the human eye no longer perceives contrast on a calibrated white-and-black target. The smaller the MOR, the denser the fog. It is considered that there is the presence of fog for a MOR below 1000 m in meteorology [30] and below 400 m in road context [31].

The fog conditions selected in the database are described in Section 3. The rest of this section shows how the numerically simulated fog is generated.

### 2.2. Numerically Simulated Fog: K-HiL Model

The numerically simulated fog on a clear weather visible image is obtained by applying a loss of contrast. The most popular method to simulate fog is to use the visibility attenuation theory of Koschmieder, defined a century ago [28]. This theory makes it possible to determine the luminance of a black object on a sky background by an attenuation of the visibility due to the extinction of the medium between the object and the observer. According to the Koschmieder law, the visibility *V* (in m) is related to the extinction coefficient $\beta_{ext}$ (in m${}^{-1}$), if we consider that the minimum contrast identifiable by an observer is 0.05 (i.e., 5%) [32].

(1) $$V=\frac{-ln(0.05)}{\beta_{ext}}$$

The transmittance of a pixel at position (x,y) in the scene is a relation between the distance $d_{x,y}$ from a target to the observer and the extinction coefficient $\beta_{ext}$ of the medium (in m${}^{-1}$) [33]:

(2) $$t_{x,y}=exp\left(-\beta_{ext}d_{x,y}\right)$$

Based on the attenuation law of Beer–Lambert [28,34], the object luminance $L_{x,y}$ of a pixel (x,y) at a distance of $d_{x,y}$ with intrinsic luminance of $L_{0;x,y}$ and $L_{s}$, being the luminance of the air light, can be described by the following relation:

(3) $$L_{x,y}=L_{0;x,y}exp\left(-\beta_{ext}\cdot d_{x,y}\right)+L_{s}\left(1-exp\left(-\beta_{ext}\cdot d_{x,y}\right)\right)$$

The depth $d_{x,y}$ from the observer to the target is used to obtain the right estimation of the transmission map, which makes it an important parameter for an accurate simulation of adverse weather on camera images.

**Figure 4:**
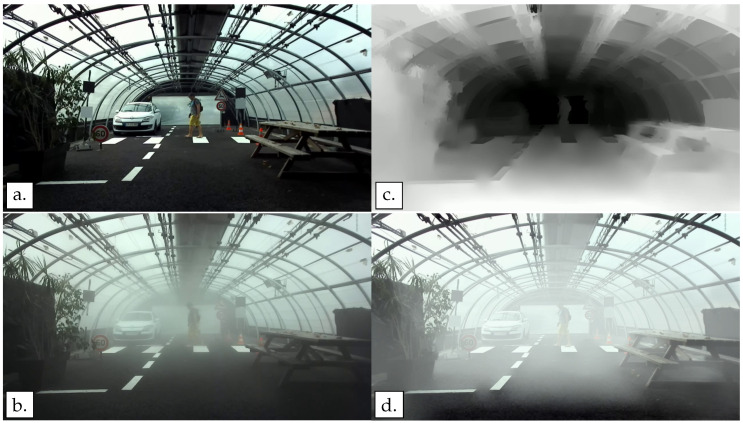
Example of an image with clear weather, real fog, and numerically simulated fog. The depth map is shown as an illustration of the stereoscopic outputs. (**a**) The initial image acquired in clear weather. (**b**) An image taken under the same conditions (pedestrian outfit and position) but with real fog generated with the PAVIN fog and rain platform. (**c**) The depth map, one of the crucial parameters for numerical fog simulation, the depth map comes from the output of the stereoscopic camera. (**d**) The corresponding image with the numerically simulated fog based on the (**a**,**c**) images data.

The equation requires three main parameters: the MOR value (*V*), the background luminance ($L_{s}$), and the depth of objects in the images ($d_{x,y}$). The depth can be extracted from the stereoscopic camera images. The visibility values depend on the artificial fog parameters from the tests. It will later be explained in Section 3. Finally, the background luminance is considered as the mean luminance of 10% of the brightest pixels of the image. Figure 4 shows an example of an image acquired in clear weather (Figure 4a) with a pedestrian crossing the crosswalk, a depth image from the stereoscopic camera (Figure 4c), an image acquired with the same pedestrian characteristics under artificial fog conditions (Figure 4b), and an image on which the fog has been numerically simulated (Figure 4d).

The two types of fog (physical and numerically simulated) are presented; the rest of this section will now explain which metrics are used in this paper, before presenting the database set up in the next section.

### 2.3. A Metric Based on a Pedestrian Detection Algorithm

As explained above, our approach to comparing and qualifying physical and numerical test equipment is based on analysis of the results obtained by a detection algorithm, rather than on analysis of the raw images themselves. To do so, it is, therefore, necessary to have a pedestrian detection algorithm, a database labeled with ground truth, and a detection algorithm evaluation metric. In this paper, we have chosen to use the AUC score.

Concerning the detection algorithm, the third version of the YOLO detection algorithm [29], which stands for “You Only Look Ones”, was chosen in this analysis. It is indeed a very common algorithm in the literature on object detection. Moreover, it is very easy to handle. The library of objects available in this version contains 80 items. The algorithm requires two main parameters: the confidence threshold (a value between 0 and 1) of the labeling and the object to label in the images. Only the class “person” is labeled in this study and the confidence threshold chosen is explained in the following section. An image in our database can obtain multiple detections with different levels of confidence even though only one pedestrian is walking in the scene in our database. As a reminder, the objective is not the evaluation of the YOLO algorithm but to use a popular object detection algorithm to evaluate the main characteristics of the database, and to compare numerical and physical artificial fogs.

In object detection, a metric widely used to evaluate the validity of a detection is the intersection over union (i.e., *IOU*) between bounding boxes. The intersection is calculated following the equation:

(4) $$IOU\left(frame\right)=\frac{\mathrm{Area}\;\mathrm{of}\;\mathrm{Overlap}}{\mathrm{Area}\;\mathrm{of}\;\mathrm{Union}}$$

**Figure 5:**
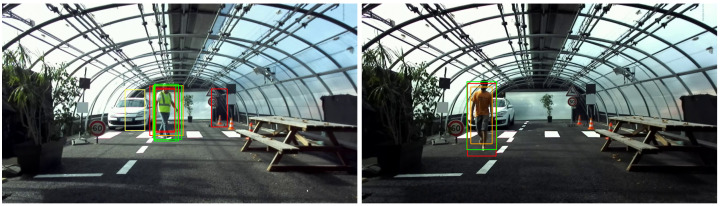
Example of YOLO detections on two clear weather images with different pedestrians. Colors: Green is for confidence >0.9, yellow is for 0.9> confidence >0.7, orange is for 0.7> confidence >0.5, Red is for 0.5> confidence >0.3.

The precision–recall curve is then calculated based on the results of intersection over union values. The curve shows the trade-off between precision and recall for different confidence threshold values from the YOLO algorithm. As an example, the different detections obtained by the YOLO algorithm, for different levels of confidence, from 0.3 to 1, on two images from the database, are presented in Figure 5. The left image of Figure 5 shows the 9 YOLO labels with two labels far from the pedestrian present in the scene, yet for one of them a confidence value greater than 0.5. The image on the right shows labels well-centered on the pedestrian, but with a high variability of the confidence value ranging from less than 0.5 to more than 0.9.

Then, the AUC score is calculated. A large AUC value represents both high recall and high precision. A high precision value indicates a low false positive rate (good confidence value but no ground truth label), and a high recall value indicates a low false negative rate (low confidence value but ground truth has a label).

The evaluation method and detection tools just presented are applied to a new pedestrian database created specifically for this study. Detailed characteristics of the Cerema Foggy-Twin Pedestrian database are presented in the next section.

## 3. The Cerema Foggy Twin Pedestrians Database

**Figure 6:**
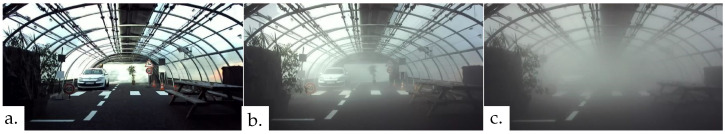
Three weather conditions for a daytime configuration of the scene with (**a**) clear weather CW, (**b**) medium fog MF, and (**c**) dense fog DF.

This section presents the main features of the Cerema Foggy-Twin Pedestrian database. This database contains real data, of different pedestrians (winter vs. summer clothes, light vs. dark clothes, accessories of different sizes, etc.), walking through an urban scene in day and night conditions, in clear weather, or in foggy conditions (Figure 6). In addition to real data, the database includes digitally simulated fog data and manual pedestrian labeling for both visible light and infrared thermal cameras. All these features are described in the following sections.

### 3.1. Real Data

The measurements were carried out in October 2022 by the ITS team of Cerema at the Cerema PAVIN fog and rain platform in Clermont-Ferrand (France). To capture the pedestrians walking through the platform, two sensors were used:

- A stereoscopic camera (ZED 2I from StereoLabs [35]) to obtain visible images and depth information. The focal lens of the camera is 4 mm, the frame rate is 19 fps, and the image size is 1280 × 720 pixels.
- A thermal camera named Serval 640 GigE from Xenics [36], with a frame rate of 25 fps, and the image size is 640 × 480 pixels.

**Figure 7:**
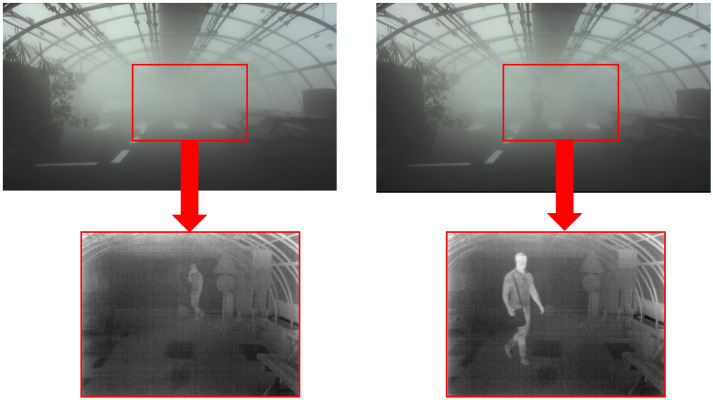
Examples of the field of view of the ZED 2I stereoscopic camera (**top** images) with a synchronized field of view of the thermal camera inside the red rectangles (**bottom** images).

**Figure 8:**
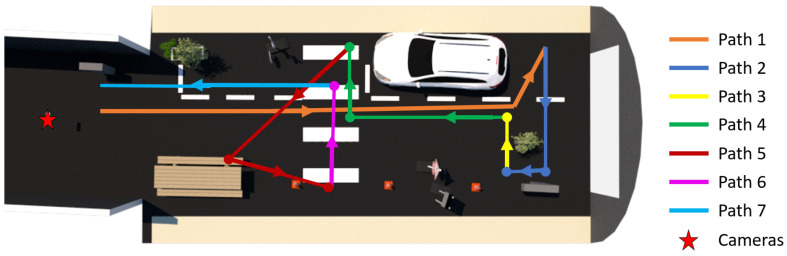
Path of the pedestrians during the tests following the colored lines and arrow directions.

The Serval camera’s field of view is smaller than that of the ZED 2I camera. The Serval camera’s position has been adjusted to cover most of the pedestrian’s path (see Figure 7). This has an impact on paths 1 and 7 (see Figure 8), which are partially or totally absent from the infrared images.

The distance or depth of each scene element relative to the cameras is extracted from the ZED 2I stereoscopic camera measurements and is part of the data available in the CFTP database. This parameter is a fundamental element in the process of numerical simulation of fog on visible images (K-HiL model). Data from both cameras are synchronized in time.

To recreate a realistic environment, an urban scene with different elements was created in the PAVIN fog and rain platform: a Renault Megane vehicle, trees, a wooden picnic table, different traffic signs, ground marking strips, and orange traffic cones, as well as four calibrated targets (a large black and a large grey (50 × 50 cm), and a small white and a small black (30 × 30 cm). A 3D model (digital twin) with all the elements of this scene is also available with the dataset.

For each trial, the pedestrians follow the same path through the platform and repeat it twice, consecutively, to ensure repeatability. Following the different colored lines in Figure 8, the path allows the pedestrian to be presented from the front (paths 4 and 7), the back (path 1) and the side (path 2, path 3, path 5, and path 6), in relation to the camera position (the red star in Figure 8). In addition to walking at a moderate pace, the pedestrians also find themselves sitting on the bench at the picnic table.

**Figure 9:**
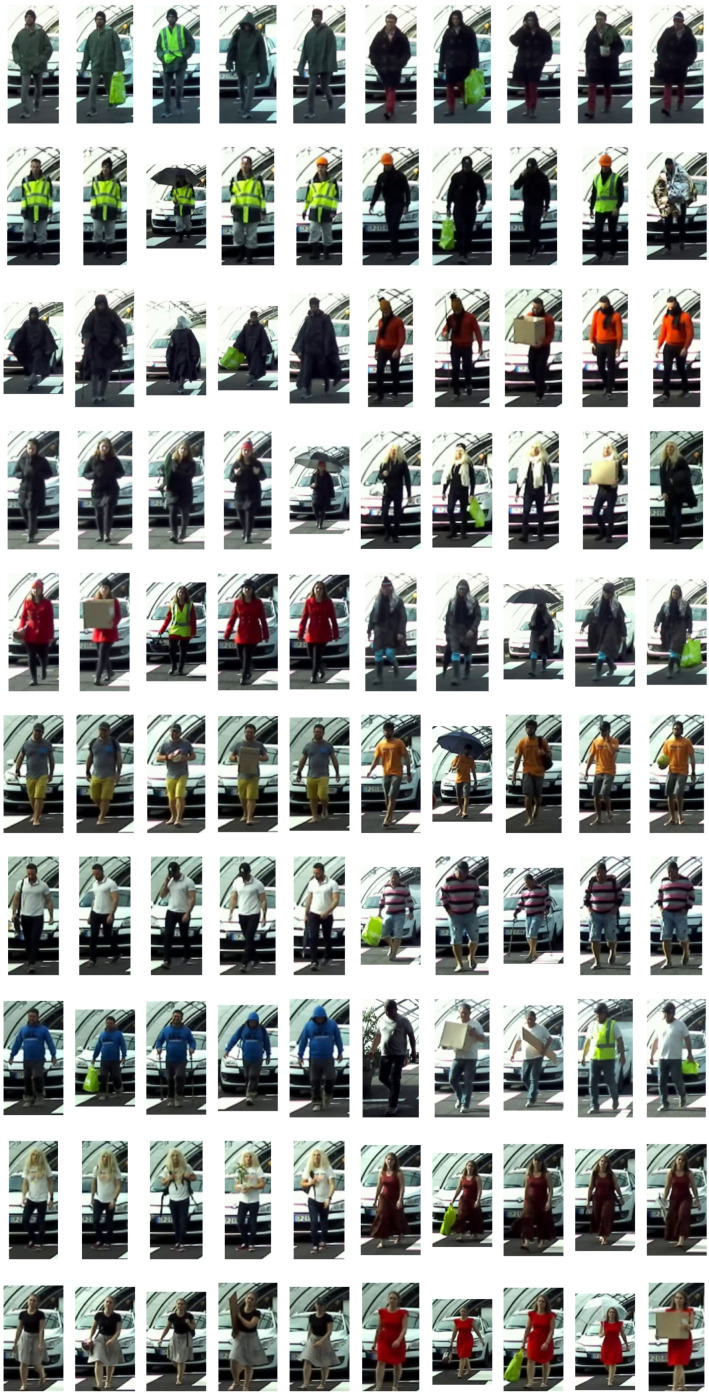
Thumbnail of the 100 pedestrians of the CFTP database.

To be representative of a wide variety of pedestrians, different characteristics have been made variable to form the batch of 100 different pedestrians (Figure 9) such as:

- Clothing: 50% of the clothing is representative of summer weather and 50% of winter weather.
- Accessories: a selection of pedestrians carry accessories with different sizes.
- Gender: 60% of the pedestrians are male and 40% are female.

Different sizes of accessories have been used in the tests. The objective is to have an impact on the overall silhouette of the pedestrian in an attempt to fool the detection algorithm. Taking into account the accessories worn by the pedestrian is crucial to guarantee his safety. An object worn by the pedestrian that would not be detected by the detection algorithm of an autonomous vehicle could endanger the pedestrian.

The data can be classified into four sub-lists:

- Small: For small accessories, such as a small backpack, a helmet, a plant, etc.
- Large: For large accessories, such as a large cardboard box, a snowboard, an open umbrella, etc.
- No accessories: When the pedestrian is not wearing any accessory or the accessory does not alter the pedestrian’s overall silhouette (e.g., a headlamp, a yellow fluorescent vest, a cell phone).
- All: All pedestrians, regardless of the accessory sizes.

Table 1 shows the distribution of the number of pedestrians by the accessory size category and a thumbnail of the 100 pedestrians in the CFTP database is shown in Figure 9.

Each pedestrian passes through each of the six defined configurations: two lighting conditions and three weather conditions. The three types of weather conditions chosen are:

- Clear weather (CW): it allows for a reference scene without disturbances due to the presence of fog.
- Medium fog (MF): with a MOR of 23 m, it allows modifying the general aspect of the objects of the scene by leaving all the elements of the visible scene detectable.
- Dense fog (DF): with a MOR of 10 m, it allows elements of the background to disappear for the stereo camera but not for the thermal camera.

These MOR values were chosen to obtain critical fog conditions. Thus, it is certain that these conditions will challenge the detection algorithm. Subsequently, the scores obtained by the latter will drop down, which will allow us to check whether the scores are similar for physical fog and numerically simulated fog. Figure 6 shows an example of the images obtained for the three weather conditions of the real data.

For each weather condition, two lighting types are considered in the PAVIN fog and rain platform:

- Daytime conditions, with the greenhouse opened on the sides to capture as much natural light as possible (See Figure 6).
- Night conditions, with the greenhouse totally closed (not presented in this paper).

Hence, the dataset of the tests includes a total of:

2 runs × 100 pedestrians × 3 weather conditions × 2 lightings = 1200 videos.

For each image, the database, therefore, contains data from two cameras (visible stereoscopic and thermal), along with the associated depth map. The database also contains pedestrians labeling data as ground truth. The labeling consists of tracing a 2D box containing the pedestrian and the accessory that he or she is carrying during the measurement, which clearly has an influence on the bounded box boundaries. The goal is to define the area that the vehicle should be able to detect and avoid. Images from the ZED 2I stereo camera were used to label the clear weather and medium fog images. Even though the images are recorded over a sequence of roughly one minute, the labeling does not take into account the moving property of the pedestrian, the labeling is conducted frame by frame. The images from the dense fog test conditions were labeled using the thermal camera images. Indeed, as can be seen in Figure 7 the pedestrian is barely detectable on the right ZED 2I visible image when located at crosswalk level and even invisible on the left ZED 2I visible image when he is at the bottom of the platform. In both cases, the pedestrian is easily detectable on the thermal images. A geometric and temporal calibration is used to label the pedestrians on the images of the ZED 2I camera and the thermal camera, as shown in Figure 7. The ground truth labeling of daytime measurements ends up with:

- 119,772 annotated images for clear weather;
- 113,630 annotated images for medium fog;
- 51,102 annotated images for dense fog;

(i.e., 233,402 annotated images thanks to the visible stereoscopic camera and 51,102 annotated images thanks to the thermal camera).

The characteristics of the database and the actual images contained in it have just been described in the previous section. As indicated at the beginning of the article, the study also focuses on the comparison between real and numerically simulated data. To this end, the K-HiL fog simulator for adding fog to initially fog-free images is described in Section 2.2. The following section presents the simulator parameters used to complete the database with images containing numerically simulated fog.

### 3.2. Numerical Simulation Parameters

**Figure 10:**
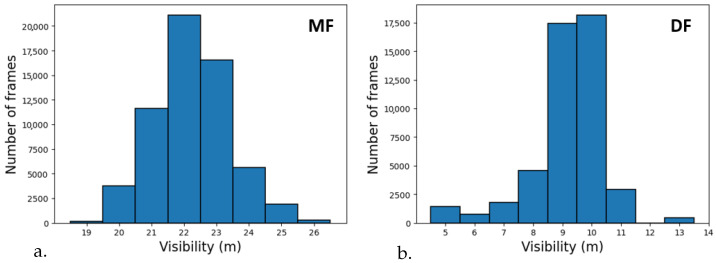
Visibility variability of MF (**a**) and DF (**b**) conditions during the tests in the PAVIN fog and rain platform.

**Figure 11:**
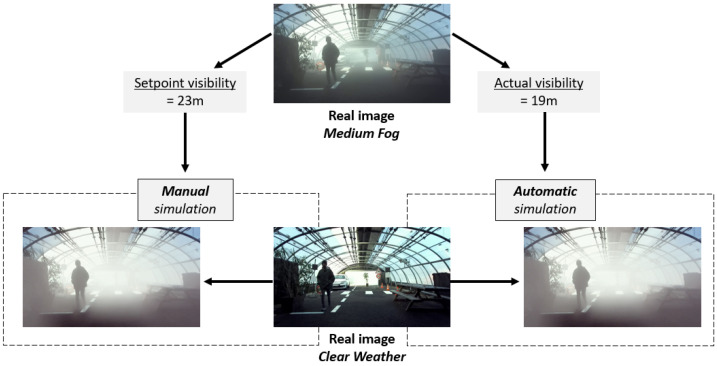
Explanation of the choice of visibility during digital fog simulation. Two modes are available for the K-HiL fog model: automatic and manual. In the two images with numerically simulated fog, the fog looks identical. However, the visibility used is 23 m in one case and 19 m in the other.

As explained in Section 2.2, the K-HiL fog simulator requires three parameters: the MOR value (*V*), the background luminance ($L_{s}$), and the depth of objects in the images ($d_{x,y}$). The depth can be extracted from the stereoscopic camera images and the background luminance is considered as the mean luminance of the 10% brightest pixels of the images. Finally, the visibility values depend on the artificial fog parameters from the tests. Figure 10 shows the distribution of visibility measured in the PAVIN fog and rain platform during the daytime tests as a histogram. Even if the visibility values have been set to 10 m for DF and 23 m for MF, a variation is observable in both cases. The histograms give a visibility of 9.1 m ± 1.15 m for DF (Figure 10b) and 21.8 m ± 0.74 m for MF (Figure 10a). The variability of visibility values in the MF tests is slightly higher with values spread over an interval between 22 and 26 m while for DF the variability is concentrated on two values, 9 and 10 m. This variability may be explained in part by the weather conditions outside the platform on the test days, which are hot and sunny, making it more difficult to stabilize fog visibility. Thus, as shown in Figure 11, two ways of taking into account this variability of visibility have been defined for the numerical simulation:

- Manual. Stable visibility fixed at 10 m for DF and 23 m for MF. These values correspond to the setpoints given for fog production on the PAVIN fog and rain platform. It means that we consider that the fog is perfectly stable.
- Automatic. Visibility that is faithful to the conditions of the production of the fog during the tests by taking the real values of visibility recorded simultaneously. As the fog varies slightly during the tests, the exact visibility recorded at the time of acquisition is used to simulate the fog numerically on the corresponding image (i.e., the same pedestrian position).

**Figure 12:**
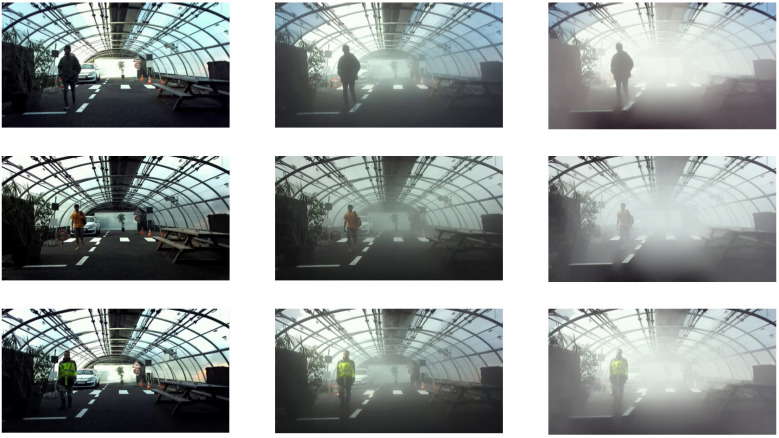
**Left:** Clear weather images, **Middle:** Artificial fog produced in PAVIN fog and rain platform, **Right**: Fog numerically simulated with visibility values of the tests. On the two right columns, from top to bottom, the medium fog visibility values are, respectively, 19, 23, and 26 m.

An example of images corresponding to three values of visibility during the MF tests is presented in Figure 12. In this figure, the three images with fog are acquired under MF conditions. This fog has a target visibility of 23 m. However, when the top row of images was acquired, the visibility of the platform was 19 m, while the visibility of the middle row was 23 m and that of the bottom row was 26 m. In automatic mode, the fog simulator, therefore, used the values 19, 23 and 26 m. In manual mode, the fog simulator would have used the value 23 m for all three images. It is almost impossible to observe this small variation of the visibility value on the three rows of fog images in Figure 12. However, it will be interesting to see whether this similarity is confirmed when the scores are evaluated. Nevertheless, if we compare the two right columns of images, we can observe a fog that appears stronger to the eye for the numerically simulated fog compared to that produced for the tests. The elements of the back of the scene are harder to identify. This visual difference can be explained by a wrong determination of the background luminance of the scene in the K-HiL model. It will also be interesting to check whether the scores obtained on the real and simulated data will be similar or different.

We, therefore, present the method and data used to meet our objectives. The following section presents the results obtained.

## 4. Results and Discussions

In this section, we will first present the main results and investigate the effects, on the scores obtained, of parameters such as weather conditions, the accessory sizes, the distance of pedestrians to the camera, and the variability of the visibility of the artificial fog from the PAVIN fog and rain platform (see Section 3.2). Thus, we will focus on the amount of data necessary for a pedestrian database, regarding parameters such as the number of frames or the number of pedestrians. Finally, we will compare the scores of both artificial fog and numerically simulated fog.

### 4.1. Global Results: Effects of Weather Conditions and Accessories

**Figure 13:**
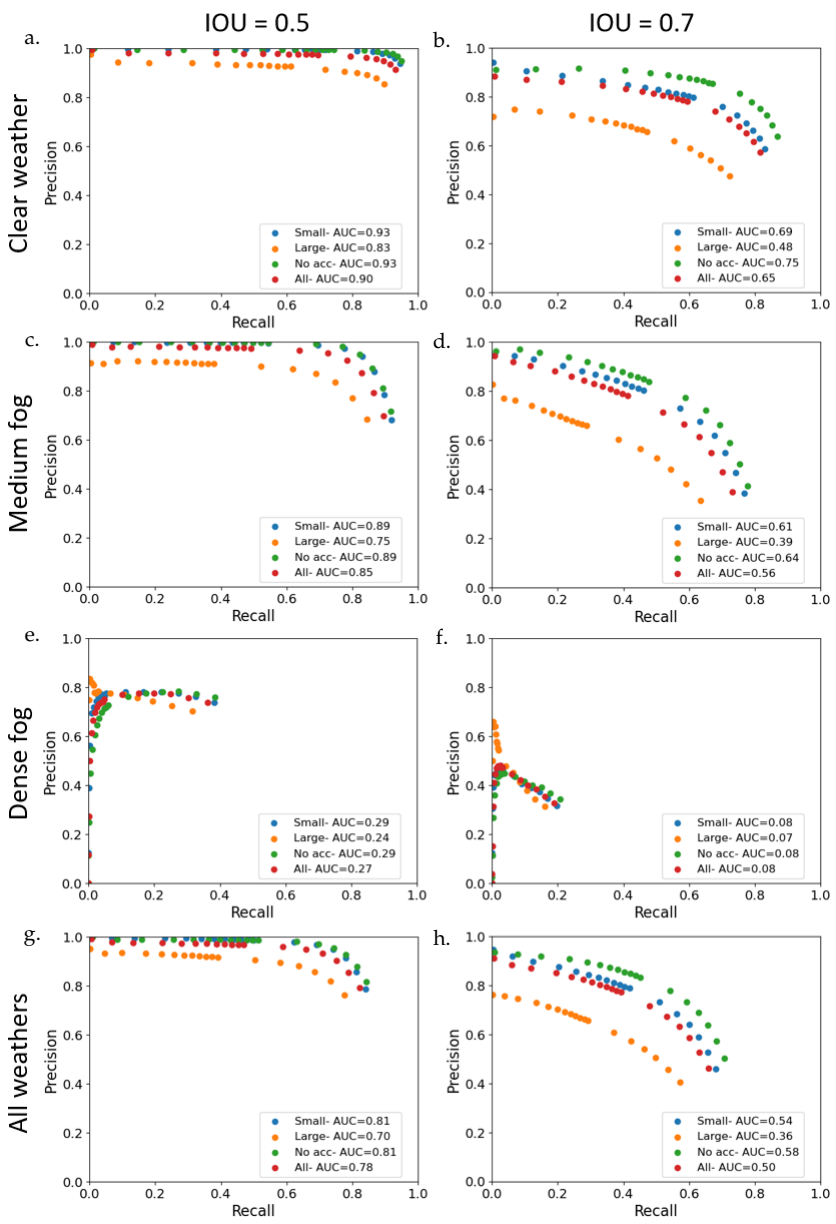
Precision and recall curves with associated AUC values for the different sub-groups based on weather conditions and accessory sizes with clear weather (**1st row:** (**a**,**b**)), medium fog (**2nd row:** (**c**,**d**)), dense fog (**3rd row:** (**e**,**f**)) and all weathers (**4th row:** (**g**,**h**)), for an IOU of 0.5 (**left column:** (**a**,**c**,**e**,**g**)) and of 0.7 (**right column:** (**b**,**d**,**f**,**h**)), and 18 values of YOLO confidence, from 0.300 to 0.999. Pedestrians with small accessories are denoted by blue dots, large accessories are denoted by orange dots, no accessories are denoted by green dots, and are denoted by red dots for the whole dataset.

After labeling the daytime images using the YOLO detection algorithm, the intersection over union (IoU) has been calculated between the YOLO labeling and the ground truth labeling. In the literature, the object detection evaluation method, like the one in the KITTI database, necessitates detection with an IoU of 0.5 for pedestrians and cyclists, compared to 0.7 for vehicles. From a relationship between the detection of true positives, false negatives, and false positives, we calculated the precision and the recall and plotted the corresponding precision and recall curves (see Figure 13) for an IOU of 0.5 and 0.7, for a continuous YOLO detection confidence from 0.300 to 0.999. Thus, the area under the curve (AUC) has been calculated for each curve corresponding to the different sub-groups of the dataset (i.e., weather conditions and accessory sizes), giving a score of YOLO labeling accuracy. Table 2 groups the scores obtained for each sub-groups. This table, therefore, presents the same data as Figure 13, but in a more summarized form. The scores of AUC in Table 2 for the IOU equal to 0.5 are stronger and closer to 1 than the scores of IOU equal to 0.7. On the curve points in Figure 13, we can hardly distinguish between cases of pedestrians without accessories or with a small accessory for an IOU equal to 0.5. On the contrary, for an IOU of 0.7, this difference can be identified with a curve for the case without accessories that stands out from the others with higher values, highlighting the impact of the presence of even a small accessory. This is why it was decided to use a more severe IOU value equal to 0.7 for the evaluation of YOLO detections in this study.

As a reminder, on the precision and recall curves (Figure 13), the highest confidence values correspond to the dots with the highest precision and lowest recall values while the dots corresponding to low YOLO detection confidence have a lower precision but a higher recall. This means that, the higher the model’s confidence value, the more accurate its detections, and the fewer false positives it detects. Conversely, the lower the confidence value, the more false positives the algorithm detects, resulting in a loss of precision. Finally, the higher the AUC score, the better the algorithm’s performance.

When comparing different weather conditions, the precision and recall curves of Figure 13 corresponding to CF, MF, and ‘All weathers’ have the best results. The precision decreases faster with lower confidence values for MF than for CW. Concerning DF, the four curves and AUC values highlight the complexity of pedestrian detection in dense fog conditions. The AUC is very low (below 0.1 for an IOU = 0.7), regardless of the type of pedestrian accessory. As previously mentioned, the hand-labeling on DF data was performed using images from the thermal camera. Indeed, in this weather condition, the pedestrians completely disappear from the visible images after crossing the crosswalk stripes, which corresponds to half the depth of the scene. Not surprisingly, labeling pedestrians beyond the crosswalk becomes complex, if not impossible, for the YOLO detection algorithm in the visible images. This is in contrast to the labeling of images with MF where the pedestrians remain visible to the back of the scene. The MOR chosen for DF (10 m) is, therefore, too dense for this type of analysis, on visible-domain sensors. Choosing the right MOR range will, therefore, be essential for testing algorithms in adverse weather conditions. In view of the results, YOLO is suitable for CW and MF conditions but is not adapted to the low visibility of DF conditions from the tests. Hence, the DF sub-dataset is not included throughout the rest of the study.

Concerning pedestrian accessories, in both cases (CW or MF), the sequences with pedestrians carrying large accessories (orange dots) obtain lower scores, demonstrating a weakness of the YOLO algorithm for this type of configuration. If we consider the scores for an IOU equal to 0.7, the best scores of AUC are encountered for the pedestrians carrying small accessories or not carrying any, giving AUC of 0.74 and 0.69, respectively, in CW conditions and 0.64 and 0.61, respectively, for MF conditions. Pedestrians carrying large accessories are challenging to detect for YOLO with scores below 0.50. Whatever the accessory size, the scores are unsurprisingly higher for CW conditions.

Therefore, accessories have a strong impact on results. Indeed, if we take the ‘No Accessories’ condition (i.e., pedestrian without accessory) as the reference, we degrade the score obtained by 7% for CW (resp. 5% for MF). When using large accessories, the score deterioration rises to 35% for CW and 39% for MF. The impact of accessories is, therefore, significant, but appears to be independent of weather conditions.

### 4.2. Effect of MOR Uncertainty on the PAVIN Fog and Rain Platform

For MF, the theoretical value of visibility (set-point) is 23 m. However, during the tests, visibility occasionally varied by a few meters as can be seen in Figure 10b. corresponding to MF tests in daytime condition. As the fog varies during the tests, it is interesting to check the impact of this variation on the results obtained by the algorithm being evaluated. The different visibility values can be grouped into sub-parts containing at least 10% of the data set: from 19 to 21 m, 22 m, 23 m, and from 24 to 26 m. Table 3 gathers for each medium fog visibility subpart of the complete dataset (i.e., all accessories included): the number of images in each subpart, the AUC value, and the relative deviation, with respect to the reference AUC (i.e., the measurements with visibility equal to 23 m). The reference AUC is the one corresponding to the frame with visibility equal to the theoretical value of visibility, i.e., an AUC equal to 0.59.

The relative difference is in the range of ±8% when the visibility values are between 22 and 26 m which represent 75% of the MF dataset. For visibility values between 19 and 21 m the relative deviation is slightly larger but does not exceed 15%. Thus, the impact of visibility variability during a fog production phase in the PAVIN fog and rain platform remains limited. This impact is not zero, however, and needs to be taken into account when estimating uncertainty.

### 4.3. Effect of Pedestrian Distance from the Camera

**Figure 14:**
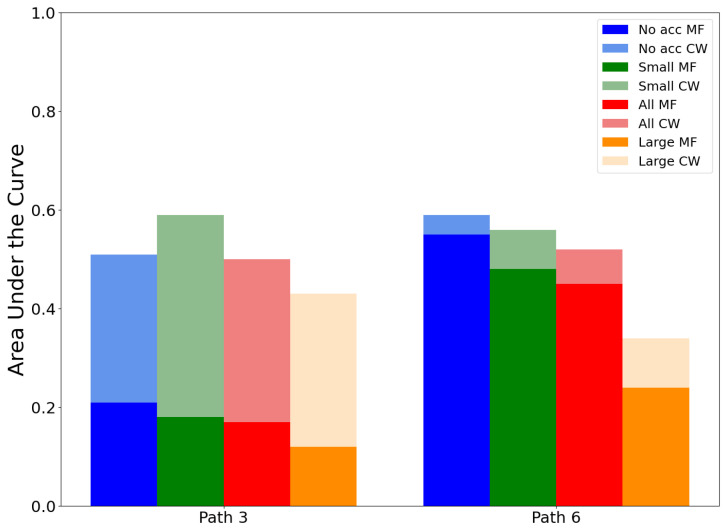
Effect of the pedestrian distance from the camera on the AUC for medium fog conditions, with light color bars, and for clear weather, with superimposed deep color bars, and accessory size subsets (no accessories in blue, small accessories in green, large accessories in orange, and all types of accessories in red).

If we now consider the effect of the distance of the pedestrian from the camera, we can investigate the scores for the frames of two perpendicular paths to the line of sight of the camera. Both paths have different depths to the camera. Path 3 is at the back of the platform, ≈18 m and path 6 to the mid-depth of the scene, ≈9.5 m (See Figure 8). The effect of the MF on the scores is clearly visible on the bar chart of path 3 (see Figure 14), although in the overall course, this difference is not very significant if we refer to the values presented in Table 2. Hence, in clear weather conditions, the distance of pedestrians to the camera does not influence the detection scores. In the case of pedestrian detection algorithm evaluation tests, it is, therefore, essential to take into account different distances between the target and sensor in foggy conditions.

### 4.4. Volume of Data Required to Create a Pedestrian Database

In this section, we aim to estimate the amount of data required for a database of pedestrians in the same urban environment to guarantee accurate and reproducible results during the evaluation of a tool, such as the YOLO pedestrian detection algorithm. This can help estimate the amount of data sufficient for a pedestrian database suitable for autonomous vehicle sensor evaluation. In this context, two factors influence the quantity of data: the number of pedestrians and the number of images (acquisition frequency) present in the database. These two elements will be evaluated in turn in this section.

Since pedestrians have different clothing, genders, and shapes, a random selection of Np pedestrians from each accessory size sub-list has been repeated 100 times with Np = [2, 5, 10, 15, 20, 25, 33, 42, 50]. The number of pedestrians per sub-list is presented in Table 1. Table 4 shows the mean AUC and standard deviation values for the 100 random selections of Np pedestrians for CW and MF conditions. The presence of accessories worn by the pedestrian, whether small or large, directly impacts the AUC score if the number of pedestrians is reduced. We find relative deviation values lower than 10% if at least 10 pedestrians are selected in the small accessory size sub-list, 25 pedestrians for the large accessory size sub-list, and only 5 pedestrians when no accessory is carried. The relative deviation is higher for CW than for MF when the pedestrian carries an accessory but lower when he does not. If we consider all the pedestrians whatever the accessory, 15 pedestrians are necessary to obtain a relative deviation below 10% for either CW or MF.

During the tests, the stereoscopic camera operated at a frame rate of 19 fps (i.e., frames per second), and the path took approximately one minute to complete, yielding just over 1000 frames. The aim here is to determine the acquisition frequency (frames per second) at which the scores converge. To achieve this, we progressively reduce the number of images in each sequence by selecting one image out of Nf, where Nf = [2, 5, 10, 20, 50, 100, 200, 400]. The operation is repeated 100 times, shifting the index of the first frame of the selection by one at each iteration. The aim here is to see whether it is worth labeling a lot of images with ground truth, or whether a sub-sample is sufficient. Table 5 groups the mean AUC values and the standard and relative deviations over the 100 iterations for the different sizes of accessory sub-lists and both CW and MF conditions.

The variability of scores when reducing the number of frames is globally limited. Nevertheless, a variation of the mean AUC value is observed when selecting 1 frame out of 50 for small and large accessory sub-lists. As mentioned in Section 3, the frame rate of the ZED 2I camera is equal to 19 fps. It is possible to select 1 frame out of 20 frames without losing information. The standard deviation becomes significant beyond 1 frame out of 20 frames. Hence, we can consider that a frame rate of 1 fps is enough to have accurate scores.

### 4.5. Intercomparison of Fog: Real Fog vs. Numerical Simulation

We will now compare the simulated fog (K-HiL model) with the real fog generated in the PAVIN fog and rain platform. Indeed, it is likely that the evaluation of AI-based algorithms will be carried out on simulated data. The realism of simulators, therefore, needs to be assessed.

From the results presented in the previous section, it has been decided that for the simulation of fog, using a noise model based on the Koschmieder law, only 1 frame out of 20 of each sequence of clear weather is processed. It allows us to reduce the amount of data without losing information. The scores are grouped in Table 6 with the AUC values of both artificial and numerically simulated medium fog. The relative deviation values are representative of the deviation of the AUC of numerically simulated fog compared to the artificial medium fog from the tests. If we first compare the two types of fog, we observe that in both cases, the scores of the numerically simulated medium fog are lower than those of the artificial fog, ranging from −7.8% to −13.1% for the “Automatic” configuration and from −6.2% to −11.5% for the “Manual” configuration depending on the accessory size. The lowest relative deviations correspond to pedestrians without accessories, while the highest relative deviations correspond to pedestrians with accessories, whether small or large. If we now compare the scores of both “Manual” and “Automatic” simulation modes, the numerical simulation with a fixed value of visibility (i.e., “Manual”) obtains lower relative deviations by 2–3 % than for the simulation with a visibility value extracted from the tests. This reinforces the negligible impact of the variability of visibility values of the fog produced within the PAVIN fog and rain platform.

Those observations are consistent with the visual analysis of the two images of simulated and real fog, made previously in Section 3.2. The numerically simulated fog is stronger than the one artificially produced. Those lowered scores mean less detection of pedestrians by YOLO for the simulated fog. Adjustments to the numerical fog simulation are necessary to move closer to the characteristics of real fog, notably by taking better account of background luminance. These results show that a simulated fog that appears denser than the real one means that pedestrians in the scene are less well detected, and, therefore, more endangered, if this detector is evaluated on the basis of the simulation and not of the real fog.

All the tests carried out on our dataset give a clearer picture of the main features needed to obtain a robust database for evaluating detection tools for autonomous vehicles. The main conclusions of this study are presented in the following section, along with some perspectives for enhancing this new database and areas of improvement in the presented tools.

## 5. Conclusions and Future Work

Vehicles featuring partially automated driving can now be certified under a guaranteed operational design domain (i.e., AEB). Artificial intelligence is mostly used for automated tasks and requires verification in all kinds of scenarios, including fog. All these qualification tests cannot be carried out in real conditions (risks or low occurrences). Thus, simulation tools for adverse weather conditions (physical and numerical) must be implemented and validated. The aim of this study is, therefore, to verify what criteria need to be met to obtain sufficient data to test AI-based pedestrian detection algorithms. It presents both analyses on data acquired in a test environment with artificially created fog conditions within the PAVIN fog and rain platform, and data from the H-KiL numerical simulator.

These data are publicly available in the Cerema Foggy-Twin Pedestrian database. They include 100 pedestrians with visible stereo images, thermal images, depth maps, manual labeling, and the digital twin of the tests, for two lighting conditions (day and night) and three weather conditions (clear weather, medium fog, and dense fog).

In order to verify which criteria are essential to obtain a good database for evaluating pedestrian detection algorithms, an original method was set up. This is based on the evaluation of a witness detection algorithm. The choice was made to use YOLOv3 in this study. In this way, the score obtained by the YOLO algorithm on different sub-sections of the database can be used to check the limits of a database in terms of ensuring a certain level of uncertainty and repeatability on the base obtained. The following parameters were taken into account in this study: weather conditions, accessory sizes, the distance of the pedestrian from the camera, variability of the visibility of the artificial fog from the PAVIN fog and rain platform, the amount of data necessary for a pedestrian database (number of frames or number of pedestrians), and artificial fog vs.numerically simulated fog. The results obtained provide a basis for validating and improving standards dedicated to the testing and approval of autonomous vehicles, including those using AI (i.e., AEB):

- The accessory sizes carried by the pedestrians influence the detection accuracy. YOLO achieves lower accuracy when the accessories are large, which could represent a risk factor for pedestrians in the case of detection dedicated to autonomous vehicle decision-making. In this field of application, detection algorithms must be able to encompass the detection of pedestrians and the accessories they wear.
- The MOR chosen for dense fog (10 m) is, therefore, too dense for this type of analysis, on visible-domain sensors. Choosing the right MOR range will, therefore, be essential to test algorithms in adverse weather conditions, but this can be a tricky task, especially as it depends on the depth of the elements in the scene. Road standards specify a maximum range of 200 m. Fog with a visibility of 10 m is far too strong for existing technologies, even though this type of fog is present in nature. This shows that the use of a passive image sensor in the visible range alone is unlikely to guarantee safety in such conditions.
- Although visibility did fluctuate slightly during the experiments inside the fog and rain PAVIN platform, it exhibited minimal influence on the scores of pedestrian detections, with approximately 8% of relative differences in the AUC values for 75% of our dataset in medium fog conditions. It shows the robustness of the platform for evaluating the performance of detection tools in foggy weather conditions. This impact is not zero, however, and needs to be taken into account when estimating uncertainty. The ability to guarantee a degree of uncertainty on the score obtained can make this type of equipment compatible with certification testing, a new and particularly remarkable result.
- The various tests carried out on the amount of data required for a robust pedestrian database enable us to estimate the minimum number of different pedestrians needed at 15, whether or not they are wearing an accessory, with less than 10% relative difference and an image acquisition frequency of around one image per second. This can obviate the need to manually label large quantities of data when it is unnecessary for achieving a low level of uncertainty. On the other hand, uncertainty can rise to around 30% if fewer than two pedestrians are used, as is the case with the main current standards (EuroNCap, AEB standard, etc.), which only use a single certified pedestrian. This calls into question the test protocols established in current standards.
- Numerical simulation of fog on clear weather images using the K-HiL model produced visually realistic data, but with a higher intensity of fog than in the images from the PAVIN platform tests. This visually observable difference is also reflected in the scores, with relative differences of around −10% between the accessory and numerical simulation configuration. One way of improving the simulation would be to take better account of background luminance, which, in our case, was estimated as the mean luminance of the 10% brightest pixels of the images. Many simulators on the market currently use this type of model, which is based on Koschmieder’s law. It is, therefore, urgent and imperative to verify and validate these simulators against real data, in order to guarantee their veracity when used as proof for certification.

The findings of this study are rich and varied, but there are still many perspectives to explore.

- To complete this study and this database of pedestrians, the night condition dataset will be analyzed. Thus, numerical simulation of fog will be performed on the nighttime clear weather images.
- In addition to this task, the numerical simulation of fog could enable us to determine the visibility value at which YOLO can no longer achieve satisfactory detection scores. This can help show the right visibility range to be tested in standards, so that the latter is compatible with technology capabilities, while being demanding enough to guarantee safety.
- Pedestrian distance has been studied here, but the database could also be used to analyze pedestrian orientation. This analysis would be highly relevant for refining the scenarios used in the standards, in order to challenge the detection algorithms to better guarantee safety.
- A hardware-in-the-loop fog model was used here, and as the digital twin is available, it would be interesting to check what the result would be for a fully virtual digital simulator (full 3D simulation).
- We chose YOLOv3 as the control algorithm in this study. However, this arbitrary choice is open to discussion, and it would be interesting to see whether the results obtained are consistent when using different pedestrian detection algorithms.

Finally, although there are still many perspectives, this study marks the start of a long process of verification and validation of testing equipment (physical or numerical) dedicated to automated driving certification.

## Figures and Tables

**Table 1:** Number of pedestrians per sub-list of accessory sizes.

Accessory Size	Number of Pedestrians
Small	25
Large	33
No Accessories	42
All	100

**Table 2:** AUC scores of YOLO pedestrian detection, depending on weather conditions and accessory sizes.

Accessories Sub-List	Weather Condition	Area Under Curve	
		**IOU = 0.5**	**IOU = 0.7**
All	CW	0.90	0.65
	MF	0.85	0.56
	DF	0.27	0.08
No Accessories	CW	0.93	0.74
	MF	0.89	0.64
	DF	0.29	0.08
Small	CW	0.93	0.69
	MF	0.89	0.61
	DF	0.29	0.08
Large	CW	0.83	0.48
	MF	0.75	0.39
	DF	0.24	0.07

**Table 3:** Impact of the variation of the visibility value during the medium fog tests in the PAVIN fog and rain platform on the AUC values and relative deviation to the AUC for frames of 23 m for MF. In bold, the reference value of 23 m.

Visibility Range (m)	Number of Frames	Area Under Curve	Relative Deviation (%)
19–21	15,527	0.5	−14.6
22	21,125	0.54	−8.0
**23**	**16,526**	**0.59**	-
24–26	7762	0.64	+8.6

**Table 4:** Effects over 100 iterations of a random selection of Np pedestrians on AUC values for clear weather and medium fog conditions.

		Accessory Size							
		**Small**		**Large**		**No Acc.**		**All**	
**Np**	**Weath. Cond.**	**Mean AUC**	**Std/Rel. (%) Deviation**	**Mean AUC**	**Std/Rel. (%) Deviation**	**Mean AUC**	**Std/Rel. (%) Deviation**	**Mean AUC**	**Std/Rel. (%) Deviation**
2	CW	0.66	0.14/22.1	0.48	0.24/49.2	0.74	0.09/ 11.6	0.62	0.17/27.9
	MF	0.61	0.10/17.1	0.39	0.17/43.0	0.63	0.11/16.9	0.54	0.16/30.4
5	CW	0.68	0.09/13.0	0.48	0.17/35.1	0.74	0.05/6.7	0.64	0.10/16.5
	MF	0.61	0.07/11.0	0.38	0.11/28.2	0.63	0.08/12.3	0.56	0.10/17.7
10	CW	0.68	0.05/7.4	0.48	0.10/20.2	0.75	0.03/4.6	0.65	0.07/11.3
	MF	0.61	0.04/6.6	0.40	0.07/17.6	0.63	0.04/6.5	0.53	0.07/13.2
15	CW	0.68	0.03/4.8	0.48	0.07/14.1	0.75	0.03/3.5	0.65	0.05/7.8
	MF	0.60	0.02/4.2	0.40	0.05/12.6	0.64	0.03/5.4	0.55	0.05/9.5
20	CW	0.69	0.02/2.8	0.48	0.05/10.9	0.75	0.02/3.0	0.64	0.05/7.6
	MF	0.60	0.02/3.1	0.39	0.03/9.1	0.64	0.03/4.3	0.56	0.04/8.0
25	CW	0.69	0	0.49	0.03/ 6.2	0.75	0.01/2.1	0.65	0.04/6.3
	MF	0.61	0	0.40	0.03/ 6.7	0.64	0.02/3.6	0.56	0.04/6.7
33	CW	-	-	0.48	0	0.75	0.01/1.3	0.64	0.04/6.0
	MF	-	-	0.40	0	0.64	0.01/2.0	0.56	0.03/6.0
42	CW	-	-	-	-	0.75	0	0.65	0.02/3.8
	MF	-	-	-	-	0.64	0	0.56	0.03/5.1
50	CW	-	-	-	-	-	-	0.65	0.02/3.9
	MF	-	-	-	-	-	-	0.55	0.02/4.0
100	CW	-	-	-	-	-	-	0.65	0
	MF	-	-	-	-	-	-	0.56	0

**Table 5:** Mean AUC, and standard and relative deviations of the AUC over 100 iterations of the selection of Nf frames for the dataset on clear weather (CW) and medium fog (MF) conditions.

		Accessory Size							
		**Small**		**Large**		**No Acc.**		**All**	
**Nf**	**Weath. Cond.**	**Mean AUC**	**Std/Rel. (%) Deviation**	**Mean AUC**	**Std/Rel. (%) Deviation**	**Mean AUC**	**Std/Rel. (%) Deviation**	**Mean AUC**	**Std/Rel. (%) Deviation**
1	CW	0.69	0.0/0.03	0.48	0.0/0.03	0.75	0.0/0.02	0.65	0.0/0.01
	MF	0.61	0.0/0.10	0.39	0.0/0.14	0.64	0.0/0.08	0.56	0.0/0.02
2	CW	0.69	0.0/0.28	0.48	0.0/0.44	0.75	0.0/0.45	0.65	0.0/0.23
	MF	0.61	0.0/0.42	0.39	0.0/0.18	0.64	0.0/0.20	0.56	0.0/0.03
5	CW	0.69	0.01/0.83	0.48	0.0/0.56	0.75	0.0/0.57	0.65	0.0/0.37
	MF	0.61	0.0/0.55	0.39	0.0/0.80	0.64	0.0/0.74	0.56	0.0/0.58
10	CW	0.69	0.01/1.18	0.48	0.01/1.56	0.75	0.01/1.27	0.65	0.0/0.82
	MF	0.61	0.01/1.51	0.39	0.01/2.35	0.64	0.01/0.95	0.56	0.0/0.76
20	CW	0.69	0.02/2.38	0.48	0.01/2.44	0.75	0.01/1.64	0.65	0.01/1.11
	MF	0.60	0.01/2.40	0.39	0.01/3.88	0.64	0.01/1.74	0.56	0.0/0.97
50	CW	0.67	0.03/4.58	0.48	0.04/7.77	0.75	0.02/2.27	0.65	0.01/2.22
	MF	0.60	0.02/3.57	0.39	0.03/7.31	0.64	0.01/2.31	0.56	0.01/2.1
100	CW	0.66	0.05/6.96	0.47	0.04/8.59	0.74	0.03/4.45	0.65	0.02/3.15
	MF	0.59	0.04/6.01	0.39	0.04/11.30	0.64	0.03/4.31	0.56	0.02/4.09
200	CW	0.67	0.06/9.03	0.45	0.05/12.16	0.74	0.05/6.23	0.65	0.02/3.94
	MF	0.61	0.04/6.58	0.34	0.05/13.96	0.63	0.03/6.56	0.56	0.03/5.14
400	CW	0.65	0.07/10.48	0.47	0.07/16.06	0.72	0.06/8.09	0.63	0.04/5.84
	MF	0.59	0.07/11.33	0.39	0.07/18.17	0.61	0.04/6.56	0.57	0.04/6.98

**Table 6:** AUC for artificial and numerically simulated medium fog, and standard and relative deviations of the AUC of artificial fog for the accessory size sub-lists.

Accessories Size	AUC			Relative Deviation	
	**Artificial Fog**	**Numerically Simulated Fog**		**Numerically Simulated Fog**	
	**(Reference)**	**Automatic**	**Manual**	**Automatic**	**Manual**
Small	0.61	0.53	0.54	−13.1 %	−11.5 %
Large	0.39	0.34	0.35	−12.8 %	−10.3 %
No Acc	0.64	0.59	0.6	−7.8 %	−6.2 %
All	0.56	0.5	0.51	−10.7 %	−8.9 %

## Data Availability

The database created within this work will soon be available online: https://ceremadlcfmds.wixsite.com/cerema-databases (accessed on 26 September 2023). If you would like to carry out your own tests on the PAVIN fog and rain platform, please contact Cerema’s STI research team at the following address: adweather@cerema.fr.

## Abbreviations

The following abbreviations are used in this manuscript:

ODD	operational design domain
AI	artificial intelligence
AV	autonomous vehicle
VRUs	vulnerable road users
ADAS	advanced driver assistance systems
AEB	automatic emergency braking
CFTP	Cerema Foggy-Twin Pedestrian
HiL	hardware-in-the-loop
AUC	area under the precision and recall curve
CW	clear weather
MF	medium fog
DF	dense fog
MOR	meteorological optical range
YOLO	you only look once
IOU	intersection over union
ITS	intelligent transportation system
